# Full-Genome Sequences of Severe Fever with Thrombocytopenia Syndrome Virus, Isolated from South Korea in 2014

**DOI:** 10.1128/genomeA.00181-15

**Published:** 2015-04-16

**Authors:** Mi-ran Yun, Sun-Whan Park, TaeSoo Kwon, SangHyun Lee, Won Gi Yoo, WooYoung Choi, Won-Ja Lee, Dae-Won Kim

**Affiliations:** a Division of Biosafety Evaluation and Control, Korea National Institute of Health, Korea Center for Disease Control and Prevention, Chungcheongbuk-do, Republic of Korea; b Division of Arboviruses, Korea National Institute of Health, Korea Center for Disease Control and Prevention, Chungcheongbuk-do, Republic of Korea; c Division of Bioterrorism Preparedness and Response, Korea Center for Disease Control and Prevention, Chungcheongbuk-do, Republic of Korea

## Abstract

Here, we present the full-length genome sequencing of severe fever with thrombocytopenia syndrome (SFTS) virus, isolated from South Korea in 2014. The five Korean strains were compared by phylogenetic analysis with full SFTS genome sequences of two neighboring nations, China and Japan.

## GENOME ANNOUNCEMENT

Severe fever with thrombocytopenia syndrome (SFTS) virus (SFTSV) is an emerging novel phlebovirus that causes SFTS, which is associated with a high mortality rate. SFTSV was first isolated in China (1) and more recently in Japan (2) and South Korea (3). The symptoms and signs caused by SFTSV infection include high fever (temperature >38°C), vomiting, diarrhea, and/or fatigue, and laboratory parameters associated with SFTSV infection are consistent with thrombocytopenia and/or leukocytopenia. SFTSV can be transmitted by various vectors, including ixodid tick species (1, 4). One study found that the prevalence of SFTSV in *Haemaphysalis longicornis* ticks, a major vector of SFTSV, was at least 0.46% in South Korea (5).

The genome of SFTSV (genus *Phlebovirus*, family *Bunyaviridae*) consists of three (L, M, and S) segmented negative-sense RNAs. These L, M, and S segments encode a viral RNA polymerase, glycoproteins (Gn and Gc), a nucleoprotein (NP), and a nonstructural S segment (NS) protein, respectively (1). In this work, we describe the sequencing of the full-length genomes of SFTSV in five strains isolated from South Korea.

These virus strains were isolated from the serum of patients who experienced high fever, vomiting, diarrhea, and fatigue in South Korea in 2014. Isolation of SFTSV was conducted as previously described (6).

The 5′- and 3′-terminal regions were determined by rapid amplification of cDNA ends (RACE) technology. The genome sequences covering all three segments were generated using *de novo* assembly with DNAStar SeqMan version 7.1 (Lasergene), and MEGA 6 was employed for the genomic sequence alignment and phylogenetic analysis using the maximum-likelihood method.

The genomes of each of the five Korean SFTSV strains (KASJH, KAGWH3, KAGBH6, KAGBH6, and KACNH3) possessed 6,368 nucleotides in segment L, 3,378 nucleotides in segment M, and 1,746 nucleotides in segment S. Full-length genome sequences within these five Korean SFTSV strains shared high similarity ranging from 96% to 99%, 93% to 99%, and 95% to 99% at the nucleotide level for segments L, M, and S, respectively.

Phylogenetic analysis was performed with the full-genome sequences of SFTSV from two neighboring Asian countries, China and Japan; seven Chinese strains (HB29, SD4, AH15, LN2, SDLZtick12, JS2012-goat01, and JS2012-tick01) and eight Japanese strains (YG1, SPL003A, SPL004A, SPL005A, SPL010A, SPL030A, SPL032A, and SPL035A) were used. Phylogenetic analysis based on S segment sequences demonstrated that all Korean strains clustered with the Chinese strains. The four Korean sequences, excluding that of strain KASJH, grouped with those of all Japanese strains, with nucleotide identities ranging from 95.8% to 99.6% and 96.3% to 99.7% for the M and L segment sequences, respectively. We may assume that these four Korean SFTSV genome sequences possibly underwent recombination.

### Nucleotide sequence accession numbers.

The full-genome sequences were deposited in GenBank as follows: strain KASJH, accession numbers KP663731 to KP663733; KAGWH3, KP663734 to KP663736; KAGBH5, KP663737 to KP663739; KAGBH6, KP663740 to KP663742; and KACNH3, KP663743 to KP663745.
